# Writing self-efficacy, achievement emotions, and engagement among Chinese EFL learners: a variable- and person-centered investigation based on control-value theory

**DOI:** 10.3389/fpsyg.2026.1895962

**Published:** 2026-09-03

**Authors:** Hong Lei, Quanwang Zhan

**Affiliations:** 1 Center for University Foreign Language Education, Anhui University, Hefei, China; 2 School of Foreign Studies, Anhui University, Hefei, China

**Keywords:** control-value theory, L2 writing emotions, L2 writing engagement, L2 writing self-efficacy, latent profile analysis

## Abstract

Drawing on the Control-Value Theory (CVT), this study adopts a dual-analytical approach to investigate the interplay of writing self-efficacy, emotions (enjoyment, anxiety, boredom), and engagement among 1,879 Chinese EFL learners. The variable-centered analysis identified writing self-efficacy as a key correlate of both behavioral and cognitive engagement, showing significant direct associations with engagement as well as indirect associations through the differentiated mediating roles of the three emotions. Notably, boredom emerged as the emotional correlate of behavioral engagement, whereas all three emotions were associated with cognitive engagement, suggesting that the role of emotions in engagement may not be uniform across engagement dimensions. Complementing these findings, the person-centered analysis identified four distinct latent profiles ranging from High Efficacy and Enjoyment to Lowest Efficacy and High Distress, which differed systematically in engagement outcomes. While behavioral engagement was comparatively insensitive to differences in emotional intensity once self-efficacy was low, cognitive engagement showed a clearer hierarchical pattern across profiles. The identification of a subgroup exhibiting concurrent high anxiety and high boredom alongside severely diminished self-efficacy illustrated a level of emotional complexity that variable-centered analyses would be unlikely to capture. Synthesizing these findings, the study suggests the potential value of considering both general associative patterns and learner-specific heterogeneity in future research and instructional practice on L2 writing.

## 1 Introduction

Learner engagement lies at the heart of effective second language acquisition (SLA), serving as a primary indicator of how deeply and productively a learner invests in the educational experience (Mercer, 2019). Accumulating evidence in the L2 field highlights that engagement is fundamentally associated with learners’ affective states: driving forces such as foreign language enjoyment stimulate persistent effort and active participation, while debilitating feelings like boredom drain cognitive energy and trigger avoidance behaviors (Derakhshan and Fathi, 2024; Saito et al., 2018; Tsang and Dewaele, 2024). However, while the intricate link between emotion and general L2 engagement has been extensively mapped, empirical exploration regarding how affect shapes investment within the specialized domain of L2 writing remains limited. Given that written composition imposes intense cognitive loads and severe self-regulatory demands, an understanding of how distinct emotional experiences shape writing engagement is critically needed.

A central psychological factor driving these emotional experiences and subsequent engagement is L2 writing self-efficacy (WSE), which reflects learners’ beliefs in their ability to manage the linguistic, performative, and self-regulatory demands of composition (Teng et al., 2018). WSE shapes learners’ affect, serving as a catalyst for positive emotions like enjoyment while buffering against negative states such as anxiety (Wang et al., 2021). However, the specific mechanisms explaining how WSE transmits its effects to writing engagement remain under-explored. In particular, empirical investigations into the mediating pathways of distinct emotions between self-efficacy and engagement are scarce within L2 writing.

The Control-Value Theory of achievement emotions (Pekrun, 2006, 2024) provides a compelling logic to explain how these predictive pathways unfold. CVT posits that academic emotions are domain-specific experiences shaped by control-value appraisals, which subsequently determine learning outcomes (Pekrun, 2024). Within this framework, L2 writing self-efficacy serves as a manifestation of control appraisal, the proximal cognitive evaluation that theoretically underlies whether the writing process is experienced with enjoyment, anxiety, or boredom, thereby being associated with engagement (Pekrun and Linnenbrink-Garcia, 2022). Drawing on this theoretical framework and the accumulating empirical evidence in L2 studies, the present study aims to scrutinize these interrelationships within the domain of L2 writing.

The present study adopts a dual-perspective methodological design to address this objective among Chinese EFL learners. We utilize variable-centered analysis (structural equation modeling) to examine the generalized, directional pathways stretching from L2 writing self-efficacy, through the mediating channels of enjoyment, anxiety, and boredom, to writing engagement. As a complementary lens, we simultaneously employ person-centered analysis (latent profile analysis) to identify distinct subgroups of learners, revealing how these mechanisms manifest as unique cognitive-affective profiles within individual students. By combining these two complementary lenses, this study aims to provide a more comprehensive understanding of the affective and motivational mechanisms underlying EFL writing engagement.

## 2 Literature review

### 2.1 The control-value theory

The theoretical foundation of the present study is grounded in the Control-Value Theory of achievement emotions (Pekrun, 2006, 2024). As an integrative framework, CVT transcends traditional approaches that viewed affect merely as a by-product of learning or focused exclusively on singular constructs like test anxiety. Instead, CVT posits that achievement emotions are ubiquitous, multifaceted phenomena that serve as determinants of learners’ learning, engagement, and performance. Central to CVT is the proposition that achievement emotions are induced by learners’ cognitive appraisals. Specifically, two categories of appraisals are deemed critical: control and value (Pekrun, 2006, 2024). Control appraisals pertain to the learner’s perceived causal influence over achievement activities and outcomes. Value appraisals refer to the subjective importance attributed to the activity or its outcome. CVT postulates that discrete emotions arise from specific combinations of these appraisals.

CVT serves as an explanatory framework that integrates the antecedents and consequences of emotion into a single, coherent model. It positions control-value appraisals as the proximal, mediating link between distal antecedents (e.g., achievement goals, growth mindset, task difficulty, autonomy support, feedback) and achievement emotions: success and high-quality, autonomy-supportive environments strengthen control and value appraisals and thus promote positive emotions, whereas failure, excessive task demands, or insufficient value undermine these appraisals and intensify negative emotions such as anxiety or boredom. On the outcome side, CVT differentiates emotions by activation and valence to predict their effects on learning—positive activating emotions (e.g., enjoyment) generally enhance performance by broadening cognitive resources and sustaining motivation, while negative deactivating emotions (e.g., boredom) are detrimental (Pekrun, 2024).

While CVT originated in general educational psychology, its application to second language acquisition is theoretically warranted by the principle of domain specificity of emotions. CVT explicitly posits that achievement emotions are not static traits but are functional responses specific to particular academic environments and subject domains (Pekrun, 2024). Recent empirical studies involving Chinese EFL learners have successfully validated the cross-contextual applicability of CVT (Li, 2024; Shao et al., 2020; Sun et al., 2026). Building on this empirical foundation, CVT provides a coherent explanatory logic for the hypothesized mediation model in the current study.

Drawing on CVT, the present study examines a theoretically grounded pathway in the context of L2 writing: from self-efficacy through writing emotions to writing engagement. Self-efficacy serves as the focal indicator of control appraisal—not an arbitrary proxy, but the construct from which CVT’s control dimension was originally derived (Pekrun, 2024)—and, crucially, represents a conceptually well-defined and firmly established construct within the field of SLA. Engagement is treated as the outcome variable, consistent with Pekrun and Linnenbrink-Garcia’s (2022) account of how achievement emotions shape engagement. While CVT centers on emotions as the mechanism linking control appraisals to outcomes, self-efficacy theory (Bandura, 1977) further suggests that efficacy beliefs may also be directly associated with engagement. The present study therefore examines both the emotion-mediated pathways proposed by CVT and the potential direct association between self-efficacy and engagement. The following subsections review empirical studies on each construct within the L2 writing domain.

### 2.2 Emotions in L2 writing

Historically, L2 emotion research was dominated by a singular focus on anxiety (MacIntyre, 2017). However, the “affective turn” in SLA, catalyzed by the introduction of Positive Psychology, has expanded the field’s horizon to encompass a wider spectrum of emotions (Dewaele and Li, 2020). Contemporary scholarship now views enjoyment, anxiety, and boredom as the “three stars orbiting each other in an unstable three-body system” (Dewaele et al., 2023, p. 7). These three emotions are ubiquitous across cultural contexts and educational levels, serving as critical indicators of learners’ psychological well-being and academic success.

Foreign language anxiety, the most extensively researched emotion, is defined as the tension and apprehension associated specifically with L2 contexts (Lou and Noels, 2020; MacIntyre and Gardner, 1994). This emotion has been found to impair learners’ fundamental cognitive processes (MacIntyre and Gardner, 1994), disrupt their self-regulation capabilities (Bown and White, 2010), and reduce their willingness to communicate (Khajavy et al., 2018). In contrast, foreign language enjoyment has emerged as a positive force. Defined as a complex state of fun, interest, and happiness, enjoyment arises when learners perceive a challenge as manageable and meaningful (Dewaele and MacIntyre, 2016; Li and Li, 2024). Empirical studies consistently position enjoyment as a positive predictor of L2 achievement, often acting as a buffer against the deleterious effects of negative emotions (Botes et al., 2022; Li et al., 2020). Foreign language boredom is a distressing experience of disengagement and dissatisfaction caused by the lack of meaningful involvement in L2 learning (Li et al., 2025). Recent meta-analytic evidence has established a negative correlation between boredom and L2 achievement (Li et al., 2025).

L2 emotions are skill-specific. Among these three key emotions, L2 writing anxiety is the most thoroughly investigated. Conceptualized by Cheng (2004) as a multidimensional construct comprising somatic anxiety, avoidance behavior, and cognitive anxiety, this emotion has been linked to detrimental outcomes such as procrastination, motivational decline, and writing paralysis (Russell-Pinson and Harris, 2019). However, empirical findings regarding the relationship between writing anxiety and achievement remain nuanced. While numerous studies report significant negative correlations (Cheng, 2004; Tahmouresi and Papi, 2021), others have found non-significant links (Rahimi and Zhang, 2019), suggesting that anxiety alone may not fully explain the variance in writing performance.

The construct of L2 writing enjoyment comprises intertwined motivational, cognitive, and affective dimensions that continuously influence one another (Jin, 2023). Theoretically, enjoyment serves as a catalyst for engagement. Bruning et al. (2013) suggested that learners who enjoyed writing possessed greater confidence in navigating the composition process and exhibited enhanced capacity for idea generation. Furthermore, Graham (2018) emphasized that positive emotional experiences facilitated the self-regulation of writing behaviors, thereby boosting performance. However, despite these assertions linking positive affect to better outcomes, empirical research specifically verifying the relationship between enjoyment and L2 writing engagement remains relatively scarce.

Parallel to the emerging interest in enjoyment, attention is also shifting toward L2 writing boredom. Li et al. (2023) identified boredom as one of the most frequently experienced emotions among Chinese EFL students and validated the Foreign Language Writing Boredom Scale. Crucially, empirical evidence from this line of inquiry points to a negative association with achievement (Li et al., 2023; Wang and Xu, 2024). Yet, despite these preliminary findings highlighting its potential impact, the specific link between L2 writing boredom and engagement remains largely under-explored.

### 2.3 L2 writing self-efficacy

L2 writing self-efficacy is conceptualized as a multidimensional construct comprising three interconnected domains (Golparvar and Khafi, 2021; Teng et al., 2018). Linguistic self-efficacy: Writers’ confidence in their ability to retrieve lexical items, employ complex syntactic structures, and adhere to discourse conventions to ensure linguistic accuracy (Sun and Wang, 2020). Performance self-efficacy: Writers’ belief in their capacity to successfully execute specific writing tasks—such as producing coherent, well-structured essays—and manage the writing process as a communicative social act (Teng et al., 2018). Self-regulatory efficacy: Writers’ confidence in their metacognitive control, specifically their ability to monitor progress, manage time efficiently, and maintain perseverance throughout the drafting process (Csizér and Tankó, 2017).

WSE functions as a primary driver of L2 writing engagement, self-regulation, and achievement (Golparvar and Khafi, 2021; Tsao, 2021). Robust efficacy beliefs foster motivational readiness and adaptability, enabling learners to deploy diverse strategies for improved outcomes (Bruning et al., 2013). Teng (2024) demonstrated that WSE significantly predicted sophisticated text processing and regulatory behaviors, such as goal-oriented monitoring and idea planning. Furthermore, Tsao (2021) highlighted the critical role of self-efficacy in facilitating learner engagement with both teacher and peer corrective feedback.

Critically, WSE plays a key role in modulating the emotional landscape of L2 writing. On the positive spectrum, learners with robust efficacy beliefs report a higher frequency of pleasant emotions, such as enjoyment and pride, alongside superior language performance (Wang et al., 2021). This positive orientation is further reinforced by the significant association between self-efficacy and perceived task value, as evidenced in integrated writing contexts (Yao et al., 2023). Conversely, WSE functions as a psychological buffer against distress. It empowers learners to surmount writing challenges by fostering resilience and adaptive coping strategies (Golparvar and Khafi, 2021; Sun and Wang, 2020). Through these mechanisms, strong efficacy beliefs effectively mitigate frustration and reduce susceptibility to negative affect and disengagement (Derakhshan and Fathi, 2024).

### 2.4 L2 writing engagement

Learner engagement is recognized as a multidimensional construct that captures the quality and intensity of learners’ involvement in learning activities (Fredricks et al., 2004; Reschly and Christenson, 2012). While definitions vary, a consensus has emerged around a tripartite framework comprising: behavioral engagement (manifested in effort, persistence, and participation), emotional engagement (reflecting learners’ affective reactions and sense of belonging), and cognitive engagement (involving mental investment and the use of deep processing or self-regulatory strategies) (Fredricks et al., 2004; Hiver et al., 2021). Research confirms that control-value appraisals significantly predict academic emotions (Li, 2021; Shao et al., 2020), which subsequently drive engagement (Liu, 2022; Sadoughi and Hejazi, 2021). Crucially, studies supporting the full mediation mechanism suggest that the impact of control-related appraisals on engagement is realized through academic emotions (King and Gaerlan, 2014).

In the specific domain of L2 writing, engagement is defined as the favorable qualities displayed by learners who are actively involved in the complex process of acquiring writing skills (Ng and Renshaw, 2023). In contrast to the abundance of general L2 engagement research, inquiry within the specific domain of L2 writing remains limited, mainly focusing on student engagement with written corrective feedback (Yu et al., 2019; Zheng and Yu, 2018) or engagement within specific writing tasks or programs (Aubrey, 2022; Namkung and Kim, 2024). To date, only a handful of studies have scrutinized the underlying mechanisms driving writing engagement. For instance, recent work has identified self-regulation as a critical mediator linking self-efficacy to engagement (Zhou et al., 2022) and validated pathways where self-regulation boosts engagement by mitigating procrastination (Zhou and Hiver, 2022). However, these investigations leave the emotional pathways bridging writing self-efficacy and engagement largely unmapped.

Despite the theoretical plausibility that emotions mediate the relationship between self-efficacy and engagement, investigations into this pathway are scarce within the specific domain of L2 writing. To bridge this gap, the present study empirically tests how L2 writing self-efficacy is associated with writing engagement through the distinct mediating roles of enjoyment, anxiety, and boredom in the Chinese EFL context. It is worth noting that enjoyment has been regarded as a constituent of affective engagement (Shernoff et al., 2014), and boredom is inherently characterized by disengagement (Pawlak et al., 2022). Given this conceptual proximity, the present study treats emotions as independent predictor constructs and thereby adopts behavioral and cognitive engagement as outcome variables to preserve discriminant validity. Furthermore, beyond the emotion-mediated pathways, efficacy beliefs may also be directly associated with engagement, so the potential direct association between self-efficacy and engagement will be examined, too.

### 2.5 The methodological synergy: combining variable- and person-centered approaches

Research in SLA has predominantly relied on variable-centered approaches, such as regression or structural equation modeling (SEM). These methods have been instrumental in validating theoretical frameworks like the CVT, advancing our understanding of the general mechanisms linking self-efficacy, emotions, and engagement across the entire population. However, while these approaches are powerful for establishing universal laws, they rely on the assumption of population homogeneity (Laursen and Hoff, 2006). By focusing on average effects, they may obscure the inter-individual heterogeneity inherent in complex learning contexts.

In reality, learners are complex individuals who often experience a “cocktail” of emotions—simultaneously feeling enjoyment and anxiety. As the proximal appraisal shaping these emotions, self-efficacy itself varies in how strongly it relates to each learner’s emotional experience. To capture this heterogeneity, person-centered approaches, specifically latent profile analysis (LPA), have emerged as a vital methodological complement to variable-centered methods in SLA emotion research (Feng et al., 2026; Sun et al., 2025; Wang and Xu, 2024). Unlike variable-centered methods, LPA classifies individuals into distinct, non-arbitrary profiles based on their shared configurations of attributes.

Critically, the present study posits that variable- and person-centered approaches are mutually reinforcing. The variable-centered approach is essential to verify the underlying theoretical mechanism of CVT—confirming how self-efficacy drives emotions and engagement. The person-centered approach is essential to reveal who the learners are and how individuals naturally cluster based on these variables.

### 2.6 The present study

Given the identified gaps, the present study combines variable- and person-centered approaches to provide a holistic understanding of the affective and motivational mechanisms underlying EFL writing engagement. This study aims to address the following research questions:

RQ1: How is writing self-efficacy associated with behavioral and cognitive engagement, both directly and indirectly through the mediating roles of enjoyment, anxiety, and boredom, among Chinese EFL learners?
RQ2: What distinct latent profiles of EFL writers can be identified based on their configurations of writing self-efficacy and achievement emotions?
RQ3: How do these identified profiles differ in their levels of behavioral and cognitive writing engagement?

## 3 Methods

### 3.1 Participants

The present study was conducted at a comprehensive university in China. Consistent with the national tertiary education curriculum, English is a compulsory subject for all non-English major undergraduate students during their first 2 years of study. Data collection was carried out in November 2025. A mixed sampling strategy, combining convenience and cluster sampling, was employed to recruit participants from a broad range of academic disciplines, including engineering, business, and the humanities. This approach ensured a representative cross-section of the non-English major population. The survey was administered via Wenjuanxing, a widely used online survey platform in China. An initial total of 1,891 responses were collected. To ensure the reliability of the data for the subsequent person-centered analysis, a rigorous data screening process was implemented. Twelve respondents were excluded due to inconsistent response patterns or insufficient completion time. The final valid sample consisted of 1,879 undergraduate students. In terms of gender distribution, the sample comprised 1,247 male students (66.365%) and 632 female students (33.635%).

### 3.2 Instruments

#### 3.2.1 L2 writing emotions

*L2 writing anxiety*. A modified version of the Second Language Writing Anxiety Inventory (Cheng, 2017) was adopted. Based on the specific context of the current study, six representative items were selected. Responses were recorded on a 5-point Likert scale ranging from 1 (strongly disagree) to 5 (strongly agree). The scale measures manifestations of anxiety such as mental blocking and apprehension (e.g., “When writing in English, I get so nervous that I go blank and cannot think clearly” and “I feel panic when I start to write in English”). In the present study, the scale demonstrated acceptable internal consistency (Cronbach’s alpha = 0.897).

*L2 writing enjoyment*. Writing enjoyment was measured using an adapted 6-item scale from Li et al. (2023). This instrument was designed to gauge students’ enthusiasm, sense of mastery, and intrinsic interest during the writing process. Participants rated their agreement on a 5-point Likert scale (1 = strongly disagree, 5 = strongly agree). Sample items include “I enjoy applying the English knowledge I have learned to my writing” and “I gain a sense of gratification from English writing.” The reliability of this scale for the current sample was robust (Cronbach’s alpha = 0.882).

*L2 writing boredom*. To capture the negative deactivating emotion of boredom, a 5-item scale was adapted from Li et al. (2023). This scale assesses students’ disengagement, lack of interest, and avoidance behaviors specifically related to L2 writing tasks. Items such as “I cannot work up any interest in English writing” and “I want to escape/avoid the task every time I write an English composition” reflect the core characteristics of writing boredom. All items were rated on a 5-point Likert scale. The scale showed satisfactory reliability in this study (Cronbach’s alpha = 0.914).

#### 3.2.2 L2 writing self-efficacy

To measure students’ beliefs in their writing capabilities, an adapted version of the Questionnaire of English Writing Self-Efficacy developed by Sun and Wang (2020) was utilized. This instrument was specifically selected for its multidimensional coverage of the writing process, capturing students’ confidence in ideation (e.g., planning and organizing), linguistic conventions (e.g., grammar and vocabulary), and self-regulation (e.g., managing emotions and persistence). The scale consists of 11 items. Participants rated their confidence levels on a 7-point Likert scale ranging from 1 (I cannot do it at all) to 7 (I can do it very well). Representative items include “I can conceive the content before I start writing,” “I can write sentences with correct grammatical structures,” and “I can persist in writing even when I encounter difficulties.” In the current study, the scale exhibited high internal consistency (Cronbach’s alpha = 0.933).

#### 3.2.3 L2 writing engagement

Student engagement in L2 writing activities was assessed using the adapted version of the Writing Engagement Scale developed by Parsons et al. (2023). Although the original scale includes an affective engagement subscale, this dimension was excluded from the present analysis due to its conceptual proximity to the emotion constructs, which were modeled as independent variables in the study. Retaining affective engagement would risk construct redundancy and compromise discriminant validity. The adapted version comprises 8 items divided into two distinct subscales: behavioral engagement (4 items), which measures the intensity of effort, concentration, and persistence exerted by students (e.g., “I stayed focused when working on this assignment” and “I worked as hard as I could on this writing assignment”); and cognitive engagement (4 items), which assesses the use of self-regulated learning strategies, such as planning, monitoring, and revising (e.g., “I asked myself questions as I was writing to make sure my writing made sense” and “I reviewed my writing and made changes to make it better”). All items were rated on a 5-point Likert scale ranging from 1 (strongly disagree) to 5 (strongly agree). In the present study, both subscales demonstrated acceptable internal consistency: Cronbach’s alpha was 0.790 for the behavioral engagement scale and 0.786 for the cognitive engagement scale.

### 3.3 Data analysis procedure

The data analysis was executed in three sequential phases using SPSS 26.0 for data management and descriptive statistics, AMOS 26.0 for structural equation modeling, and R (Version 4.3.0) for latent profile analysis.

In the preliminary phase, data were screened for normality and outliers. Given the cross-sectional, self-reported nature of the dataset, Harman’s single-factor test was performed to assess the potential threat of common method bias (CMB), ensuring that method variance did not unduly influence the results (Podsakoff et al., 2003). Subsequently, the psychometric properties of the measurement scales were rigorously validated via confirmatory factor analysis (CFA) using AMOS. Model fit was evaluated against established benchmarks, including the chi-square to degrees of freedom ratio (χ^2^/*df*), the comparative fit index (CFI), the Tucker-Lewis index (TLI), and the root mean square error of approximation (RMSEA) (Hair et al., 2019). Convergent validity was further established by examining standardized factor loadings, composite reliability (CR), and average variance extracted (AVE), while discriminant validity was verified using the Fornell-Larcker criterion (Fornell and Larcker, 1981).

To address the first research question regarding the interrelationships among variables, a structural equation model was constructed in AMOS. Writing self-efficacy was modeled as the exogenous predictor, achievement emotions as parallel mediators, and behavioral and cognitive engagement as the outcomes. To test whether the mediation was full or partial, direct paths from self-efficacy to both engagement dimensions were also included in the model. The fit of the structural model was assessed using the aforementioned indices. The significance of the indirect effects of anxiety, enjoyment, and boredom as mediators was tested using bootstrapping with 5,000 resamples to generate 95% bias-corrected confidence intervals (Hayes, 2009).

To address the second research question and identify distinct subgroups of learners, a latent profile analysis was conducted using the tidyLPA package in R (Rosenberg et al., 2018). Prior to profiling, all indicator variables were standardized (*z*-scores) to eliminate bias arising from the different metric scales (i.e., the 7-point efficacy scale versus the 5-point emotion scales). A series of models ranging from one to *k* profiles were estimated iteratively. The determination of the optimal number of profiles relied on a holistic evaluation of statistical fit and theoretical interpretability (Marsh et al., 2009). We prioritized lower values on the Akaike information criterion (AIC), Bayesian information criterion (BIC), and sample-size-adjusted BIC (SABIC), alongside a significant *p*-value (< 0.05) on the bootstrap likelihood ratio test (BLRT) (Ferguson et al., 2020; Nylund et al., 2007). Furthermore, classification certainty was assessed using entropy values (> 0.80), and the stability of the solution was ensured by verifying that the smallest profile comprised no less than 5% of the total sample (Ferguson et al., 2020).

Finally, to examine how the identified profiles differed in terms of writing engagement (RQ3), a one-way multivariate analysis of variance (MANOVA) was conducted, with latent profile membership serving as the independent variable. Upon confirming a significant multivariate effect, univariate ANOVAs were performed for both behavioral and cognitive engagement dimensions. Crucially, to account for the likely unequal sample sizes and variances across the identified profiles, Games-Howell post-hoc tests were utilized for all pairwise comparisons.

## 4 Results

### 4.1 Preliminary analyses and measurement model

Harman’s single-factor test was conducted to assess potential common method bias. The analysis extracted five factors with eigenvalues greater than 1.0, cumulatively accounting for 64.583% of the total variance. Crucially, the first unrotated factor explained only 39.740%, falling below the 50% threshold (Podsakoff et al., 2003), suggesting that no single factor accounted for the majority of variance among the study variables and common method bias was unlikely to be a serious concern in the present study.

The measurement model demonstrated satisfactory psychometric properties, as shown in Table 1. All standardized factor loadings were statistically significant (*p* < 0.001) and ranged from 0.550 to 0.885, exceeding the recommended threshold of 0.50 (Hair et al., 2019). Cronbach’s alpha values ranged from 0.786 to 0.933, and CR values ranged from 0.787 to 0.935, both surpassing the acceptable threshold of 0.70. AVE values ranged from 0.508 to 0.671 for most constructs, with the only exception being cognitive engagement (AVE = 0.480). Together, these indices provided overall evidence of convergent validity. Regarding model fit, the CFI (0.890) and TLI (0.880) approached the recommended threshold of 0.90, and the RMSEA (0.069) fell below the acceptable threshold of 0.08, indicating reasonable model fit. The χ^2^/*df* ratio (9.817) was elevated, which is commonly observed in studies with large sample sizes (Hair et al., 2019).

**Table 1:** Results for factor loadings, reliability, and convergent validity.

Dimension	Item	Unstandardized estimate	*SE*	*z*	*p*	Standardized estimate	Cronbach’s alpha	AVE	CR
L2 writing self-efficacy	1	1.000				0.734	0.933	0.569	0.935
	2	0.984	0.029	33.397	***	0.762			
	3	1.091	0.030	36.149	***	0.820			
	4	1.160	0.030	38.163	***	0.862			
	5	1.084	0.029	37.257	***	0.843			
	6	1.104	0.031	35.125	***	0.798			
	7	1.043	0.030	35.017	***	0.796			
	8	0.971	0.030	32.201	***	0.737			
	9	1.004	0.033	30.643	***	0.703			
	10	0.975	0.037	26.627	***	0.616			
	11	0.810	0.033	24.686	***	0.573			
L2 writing enjoyment	1	1.000				0.586	0.882	0.562	0.882
	2	0.968	0.040	24.460	***	0.550			
	3	1.519	0.060	25.355	***	0.754			
	4	1.675	0.061	27.402	***	0.856			
	5	1.673	0.060	27.904	***	0.885			
	6	1.614	0.061	26.339	***	0.801			
L2 writing anxiety	1	1.000				0.659	0.897	0.583	0.893
	2	1.148	0.030	37.778	***	0.726			
	3	1.054	0.037	28.268	***	0.753			
	4	1.337	0.044	30.352	***	0.824			
	5	1.214	0.042	28.632	***	0.765			
	6	1.267	0.041	30.817	***	0.841			
L2 writing boredom	1	1.000				0.856	0.914	0.671	0.910
	2	1.016	0.022	46.136	***	0.840			
	3	0.720	0.022	32.681	***	0.673			
	4	0.981	0.021	47.774	***	0.858			
	5	1.052	0.022	47.357	***	0.854			
L2 writing behavioral engagement	1	1.000				0.567	0.790	0.508	0.803
	2	1.120	0.051	21.901	***	0.712			
	3	1.264	0.054	23.295	***	0.819			
	4	1.222	0.055	22.202	***	0.730			
L2 writing cognitive engagement	1	1.000				0.699	0.786	0.480	0.787
	2	0.887	0.036	24.835	***	0.674			
	3	0.918	0.037	25.073	***	0.682			
	4	1.060	0.041	26.048	***	0.716			

*** *p* < 0.001.

Discriminant validity was assessed using the Fornell-Larcker criterion (Fornell and Larcker, 1981), as shown in Table 2. The square roots of AVE for all constructs exceeded their inter-construct correlations, with the exception of the correlation between enjoyment and boredom (*r* = −0.803), which slightly exceeded the AVE square root of enjoyment (0.750). This pattern is theoretically expected given that these two constructs represent conceptually opposing emotional valences. Overall, the measurement model demonstrated adequate validity and reliability to support subsequent structural analysis.

**Table 2:** Results for discriminant validity.

Dimension	Cognitive engagement	Behavioral engagement	Boredom	Anxiety	Enjoyment	Self-efficacy
Cognitive engagement	0.693					
Behavioral engagement	0.571	0.713				
Boredom	−0.469	−0.328	0.819			
Anxiety	−0.318	−0.247	0.664	0.764		
Enjoyment	0.561	0.308	−0.803	−0.560	0.750	
Self-efficacy	0.529	0.288	−0.562	−0.603	0.696	0.754

Values on the diagonal are the square roots of AVE.

### 4.2 Test of path relationships

The results indicated that the structural model achieved an acceptable fit to the data, yet not ideal (χ^2^/*df* = 12.131, CFI = 0.860, TLI = 0.848, RMSEA = 0.077). Although the χ^2^/*df* ratio exceeded the threshold of 5.0, this metric is known to be highly sensitive to large sample sizes (Hair et al., 2019).

The standardized path coefficients and significance levels for the structural relationships are summarized in Table 3. Regarding the paths from writing self-efficacy to the three emotions, writing self-efficacy was significantly and positively associated with enjoyment (*β* = 0.727, *p* < 0.001), and significantly and negatively associated with both anxiety (*β* = −0.628, *p* < 0.001) and boredom (*β* = −0.610, *p* < 0.001).

**Table 3:** Path relationship test results.

Path	Unstandardized estimate	*SE*	*z*	*p*	Standardized estimate
Self-efficacy → enjoyment	0.443	0.023	19.096	***	0.727
Self-efficacy → anxiety	−0.516	0.028	−18.647	***	−0.628
Self-efficacy → boredom	−0.692	0.033	−20.666	***	−0.61
Enjoyment → behavioral engagement	0.063	0.037	1.711	0.087	0.068
Anxiety → behavioral engagement	0.009	0.024	0.383	0.701	0.013
Boredom → behavioral engagement	−0.104	0.017	−6.124	***	−0.209
Enjoyment → cognitive engagement	0.369	0.048	7.627	***	0.293
Anxiety → cognitive engagement	0.134	0.03	4.461	***	0.143
Boredom → cognitive engagement	−0.102	0.021	−4.881	***	−0.15
Self-efficacy → behavioral engagement	0.078	0.029	2.685	0.007	0.139
Self-efficacy → cognitive engagement	0.255	0.038	6.769	***	0.332

*** *p* < 0.001.

Regarding the paths from emotions to behavioral engagement, only boredom was significantly and negatively associated with behavioral engagement (*β* = −0.209, *p* < 0.001). The paths from enjoyment (*β* = 0.068, *p* = 0.087) and anxiety (*β* = 0.013, *p* = 0.701) to behavioral engagement were both non-significant. Regarding the paths from emotions to cognitive engagement, all three emotions showed significant associations: enjoyment was positively associated with cognitive engagement (*β* = 0.293, *p* < 0.001), anxiety was positively associated with cognitive engagement (*β* = 0.143, *p* < 0.001), and boredom was negatively associated with cognitive engagement (*β* = −0.150, *p* < 0.001). Notably, enjoyment demonstrated the strongest association with cognitive engagement among the three emotions.

Regarding the direct paths from writing self-efficacy to engagement, self-efficacy was significantly and positively associated with both behavioral engagement (*β* = 0.139, *p* = 0.007) and cognitive engagement (*β* = 0.332, *p* < 0.001), indicating that the mediation was partial rather than complete.

### 4.3 Mediation analysis

To examine the indirect mechanisms linking writing self-efficacy to engagement, mediation analyses were conducted using the bootstrapping method with 5,000 resamples (Hayes, 2009). The significance of the indirect effects was determined based on whether the 95% confidence intervals (CI) excluded zero. The results of the mediation analysis are presented in Table 4.

**Table 4:** Results of the bootstrapping analysis for the mediation effects.

Path relationship	Point estimate	*SE*	Bootstrapping 5,000 times 95% CI			
			Bias-corrected		Percentile	
			Lower	Upper	Lower	Upper
Self-efficacy → enjoyment → behavioral engagement	0.028	0.029	−0.026	0.085	−0.027	0.085
Self-efficacy → enjoyment → cognitive engagement	0.164	0.035	0.095	0.229	0.097	0.23
Self-efficacy → anxiety → behavioral engagement	−0.005	0.017	−0.037	0.026	−0.037	0.026
Self-efficacy → anxiety → cognitive engagement	−0.069	0.022	−0.114	−0.028	−0.112	−0.027
Self-efficacy → boredom → behavioral engagement	0.072	0.021	0.034	0.115	0.033	0.112
Self-efficacy → boredom → cognitive engagement	0.07	0.028	0.019	0.125	0.017	0.121

Regarding the mediation via enjoyment, the indirect effect on behavioral engagement was non-significant, as the confidence interval included zero. However, enjoyment served as a significant positive mediator for cognitive engagement (indirect effect = 0.164), indicating that higher self-efficacy was associated with greater enjoyment, which in turn was associated with higher cognitive engagement.

Regarding the mediation via boredom, both indirect effects were significant and positive in direction: behavioral engagement (indirect effect = 0.072) and cognitive engagement (indirect effect = 0.070). Although the path from self-efficacy to boredom was negative (*β* = −0.610) and the path from boredom to engagement was also negative (*β* = −0.209 for behavioral; *β* = −0.150 for cognitive), the resulting indirect effects were positive, indicating that higher self-efficacy was associated with lower boredom, which in turn was associated with higher engagement.

Regarding the mediation via anxiety, the indirect effect on behavioral engagement was non-significant. However, anxiety served as a significant mediator for cognitive engagement (indirect effect = −0.069). This negative indirect effect reflects the fact that while self-efficacy reduced anxiety (*β* = −0.628), anxiety itself was positively associated with cognitive engagement (*β* = 0.143). Consequently, higher self-efficacy was associated with lower anxiety, which in turn was associated with lower cognitive engagement through this pathway.

In terms of relative contribution to cognitive engagement, enjoyment accounted for the largest proportion of the total absolute indirect effect (54.1%), followed by boredom (23.1%) and anxiety (22.8%), suggesting that enjoyment may represent the most prominent mediating pathway for cognitive engagement. For behavioral engagement, only the boredom pathway reached significance.

### 4.4 Latent profile analysis

#### 4.4.1 Model selection

To identify distinct subgroups of students based on their writing self-efficacy and emotions, latent profile analysis was conducted. Models with one to six profiles were estimated and compared. Table 5 presents the fit indices for the estimated models. As the number of profiles increased, the information criteria (AIC, BIC, SABIC) consistently decreased, indicating improved model fit. The BLRT results were significant (*p* = 0.01) across all models, suggesting that adding profiles provided a statistically better fit than models with fewer profiles. The four-profile solution was selected as the optimal model based on a balance of statistical fit, parsimony, and latent class interpretability. While the three-profile solution exhibited the highest entropy (0.842), the four-profile solution demonstrated superior fit indices (lower AIC and BIC). Crucially, the decision to retain the four-profile solution was driven by class size considerations. The five-profile solution resulted in a subgroup comprising only 2.7% of the sample, which falls below the recommended 5% threshold. In contrast, the four-profile solution maintained substantial class sizes, with the smallest profile accounting for 6.7% of the sample. Although the entropy (0.789) was slightly lower, considering other fit indices and the identification of substantively meaningful subgroups with sufficient sample sizes, the four-profile model was determined to be the optimal solution.

**Table 5:** Fit indices for latent profile analysis models with one to six profiles.

Model	Log-likelihood	AIC	BIC	SABIC	Entropy	BLRT *p*-value	Smallest profile %
One-profile	−10662.741	21341.483	21385.791	21360.375	1.000		
Two-profile	−9771.101	19568.202	19640.203	19598.902	0.695	0.010	48.2
Three-profile	−9174.413	18384.827	18484.520	18427.334	0.842	0.010	12.8
Four-profile	**−8964.989**	**17975.977**	**18103.363**	**18030.292**	**0.789**	**0.010**	**6.7**
Five-profile	−8857.138	17770.276	17925.354	17836.398	0.791	0.010	2.7
Six-profile	−8802.211	17670.422	17853.192	17748.352	0.784	0.010	2.5

The bold values in this table highlight the selected model (the four-profile model).

#### 4.4.2 Characteristics of the latent profiles

**Figure 1:**
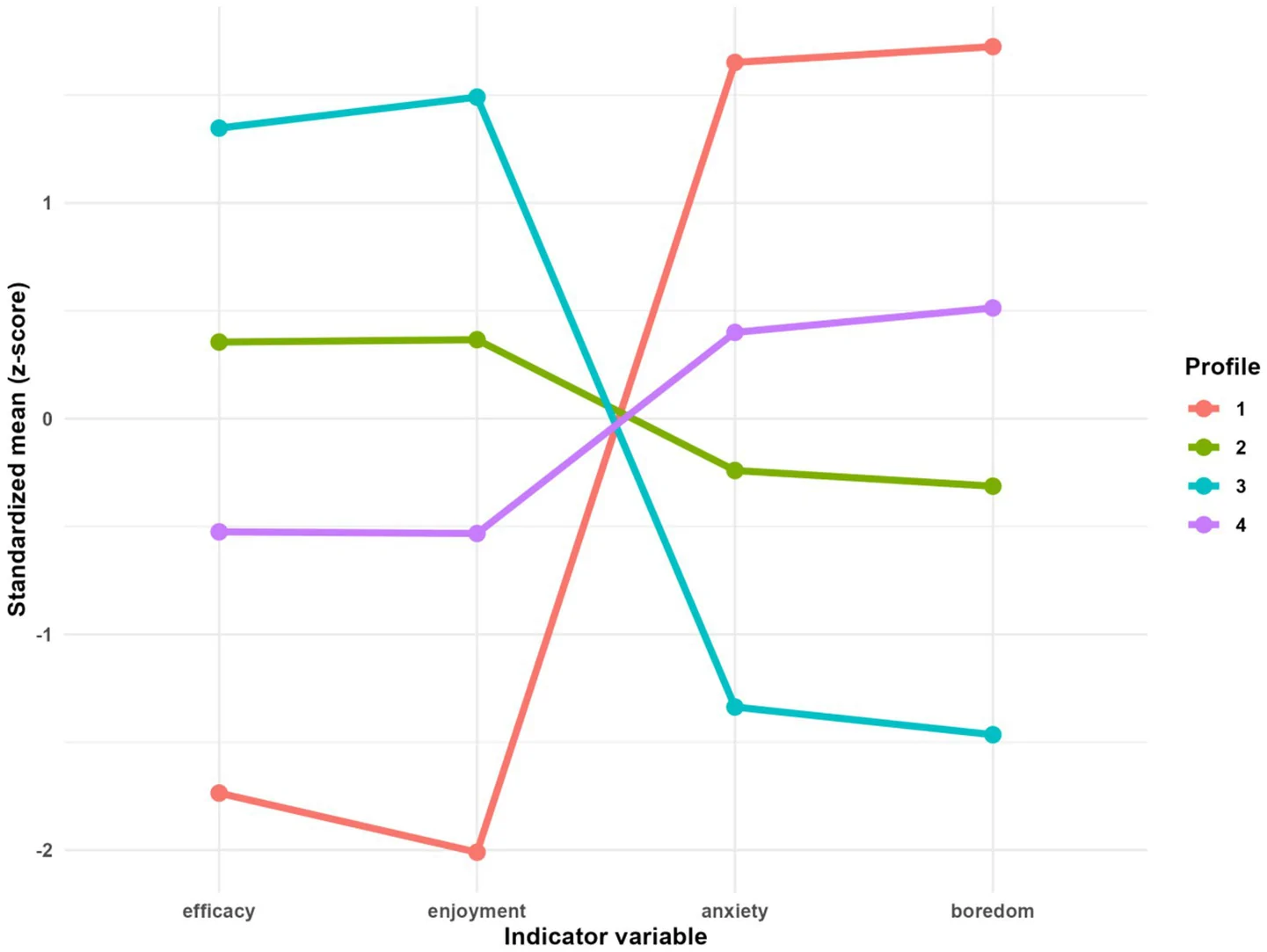
Standardized mean scores of the four indicator variables for the four latent profiles.

Based on the distinct patterns observed across the four indicators—writing self-efficacy, enjoyment, anxiety, and boredom—the identified profiles were labeled accordingly (see Table 6 and Figure 1).

**Table 6:** Descriptive statistics for the four latent profiles.

Statistics	Profile 1	Profile 2	Profile 3	Profile 4
Profile label	Lowest efficacy and high distress	Moderate efficacy and enjoyment	High efficacy and enjoyment	Low efficacy and moderate negative affect
Sample size (n)	124	838	206	711
Sample proportion (%)	6.599	44.598	10.963	37.839
Self-efficacy M (SD)	2.649 (1.015)	4.771 (0.572)	5.773 (0.612)	3.835 (0.594)
Enjoyment M (SD)	1.737 (0.453)	3.459 (0.340)	4.254 (0.422)	2.779 (0.379)
Anxiety M (SD)	4.185 (0.744)	2.614 (0.568)	1.711 (0.582)	3.163 (0.549)
Boredom M (SD)	4.276 (0.730)	2.521 (0.509)	1.550 (0.534)	3.259 (0.488)

*Profile 3*: High Efficacy and Enjoyment Profile (10.963%). The first subgroup (*n* = 206) represented the most adaptive profile. Students in this group were characterized by the highest levels of self-efficacy (*M* = 5.773) and enjoyment (*M* = 4.254), coupled with the lowest levels of negative emotions (anxiety: *M* = 1.711; boredom: *M* = 1.550). This profile reflects students who are highly confident, intrinsically motivated, and emotionally positive toward writing.

*Profile 2*: Moderate Efficacy and Enjoyment Profile (44.598%). The second and largest subgroup (*n* = 838) exhibited a generally adaptive but less intense pattern. These students reported moderate-to-high levels of self-efficacy (*M* = 4.771) and enjoyment (*M* = 3.459). While their negative emotions remained low (anxiety: *M* = 2.614; boredom: *M* = 2.521), their positive affect was not as robust as the first group, representing the “average” functioning students.

*Profile 4*: Low Efficacy and Moderate Negative Affect Profile (37.839%). The third subgroup (*n* = 711) marked a distinct transition to maladaptive functioning. Students in this group reported low levels of self-efficacy (*M* = 3.835) and enjoyment (*M* = 2.779). Crucially, unlike the first two profiles, these students manifested moderate levels of negative affect, with elevated boredom (*M* = 3.259) and anxiety (*M* = 3.163). This pattern suggests a large group of students who lack confidence and are moderately disengaged.

*Profile 1*: Lowest Efficacy and High Distress Profile (6.599%). The final subgroup (*n* = 124) comprised the most vulnerable students. They exhibited the lowest levels of self-efficacy (*M* = 2.649) and enjoyment (*M* = 1.737) across all groups. Furthermore, they reported the highest levels of boredom (*M* = 4.276) and anxiety (*M* = 4.185). The term “High Distress” implies a severe maladaptive state where students are simultaneously overwhelmed by anxiety and detached through boredom, indicating a critical need for intervention.

#### 4.4.3 Differences in L2 writing engagement across profiles

To examine whether the identified latent profiles differed in their L2 writing engagement, a one-way multivariate analysis of variance was conducted. The four profiles were entered as the independent variable, while the two dimensions of engagement (behavioral and cognitive) served as dependent variables. The MANOVA results revealed a statistically significant multivariate effect of profile membership on engagement, Pillai’s trace = 0.257, *F*(6, 3,750) = 92.13, *p* < 0.001.

Follow-up univariate ANOVAs confirmed significant differences across profiles for both engagement dimensions: behavioral engagement, *F*(3, 1875) = 106.09, *p* < 0.001; and cognitive engagement, *F*(3, 1875) = 183.98, *p* < 0.001 (Table 7). Games-Howell post-hoc tests were performed to examine pairwise differences. The results demonstrated a clear hierarchical pattern consistent with the adaptability of the profiles. The High Efficacy and Enjoyment Profile (Profile 3) exhibited the highest levels of engagement across both dimensions, scoring significantly higher than all other groups (*p* < 0.001). The Moderate-Efficacy and Enjoyment Profile (Profile 2) followed, showing significantly lower engagement than Profile 3 but significantly higher engagement than the two maladaptive profiles (*p* < 0.001). The maladaptive groups consistently reported lower engagement. Specifically, the Low Efficacy and Moderate Negative Affect Profile (Profile 4) reported significantly higher cognitive engagement than the Lowest Efficacy and High Distress Profile (Profile 1) (*p* < 0.05). However, regarding behavioral engagement, the difference between these two profiles was not statistically significant (*p* = 0.052), suggesting that both groups exhibited similarly low levels of behavioral effort.

**Table 7:** Mean scores and ANOVA results for writing engagement across the four profiles.

Engagement dimension	Profile 1 (*n* = 124)	Profile 2 (*n* = 838)	Profile 3 (*n* = 206)	Profile 4 (*n* = 711)	*F*
Behavioral engagement M (SD)	3.577 (0.737)^a^	4.001 (0.49)^b^	4.394 (0.498)^c^	3.755 (0.513)^a^	106.09***
Cognitive engagement M (SD)	2.889 (0.937)^a^	3.542 (0.549)^b^	4.062 (0.611)^c^	3.149 (0.517)^d^	183.98***

***p < 0.001. Means in the same row with different superscripts are significantly different at *p* < 0.05.

## 5 Discussion

### 5.1 Examining the CVT pathways in Chinese EFL writing

The variable-centered findings provide empirical support for the cross-contextual applicability of CVT within the Chinese EFL writing context (Li, 2024; Shao et al., 2020). Specifically, writing self-efficacy was positively associated with enjoyment and negatively associated with both anxiety and boredom (Wang et al., 2021). Furthermore, the significant direct paths from self-efficacy to both behavioral and cognitive engagement suggest that self-efficacy is associated with engagement directly beyond its effects through emotional pathways, supporting a partial mediation model. This pattern is consistent with Bandura’s (1977) proposition that efficacy beliefs shape behavioral investment, and may reflect the particular salience of writing self-efficacy in the Chinese EFL context.

The mediation analysis reveals that self-efficacy is associated with engagement through distinct emotional pathways that differ across engagement dimensions. Regarding behavioral engagement, only the boredom pathway reached significance. A stronger sense of writing competence appears to be associated with reduced boredom, which in turn is associated with higher behavioral engagement. Within the CVT framework, boredom is classified as a negative deactivating emotion characterized by low arousal and disengagement (Pekrun, 2006); its reduction therefore appears to be a prerequisite for sustaining the effortful, persistent behaviors that constitute behavioral engagement in Chinese EFL writing classrooms, where students may otherwise disengage from perceived meaningless or repetitive writing tasks. The non-significant indirect paths via enjoyment and anxiety suggest that for behavioral investment, the mitigation of deactivating affect may matter more than the presence of positive or activating emotions.

Regarding cognitive engagement, all three emotions functioned as significant mediators, albeit in different ways. Enjoyment accounted for the largest proportion of the total absolute indirect effect on cognitive engagement, suggesting that self-efficacy may facilitate deeper cognitive processing primarily by fostering intrinsic interest and positive affect toward writing. This aligns with the Broaden-and-Build Theory (Fredrickson, 2001), which posits that positive affect broadens cognitive repertoires, encouraging learners to invest more effort and use deeper strategies.

Most intriguingly, anxiety emerged as a significant negative mediator of cognitive engagement: higher self-efficacy was associated with lower anxiety, which in turn was associated with lower cognitive engagement through this pathway. This finding is consistent with CVT’s classification of anxiety as a negative activating emotion characterized by high arousal, which may under certain conditions mobilize cognitive effort (Pekrun, 2024). In the Chinese EFL writing context, moderate anxiety may prompt learners to monitor their writing more carefully and employ self-regulatory strategies more actively. This nuanced finding highlights that the relationship between anxiety and cognitive engagement is not straightforwardly negative, and suggests that intervention strategies targeting anxiety reduction should be carefully calibrated.

### 5.2 Unveiling population heterogeneity

While the variable-centered findings empirically support the sequential logic of the Control-Value Theory: control appraisals (i.e., self-efficacy) are associated with affective experiences, which subsequently regulate the quality of engagement, the person-centered analysis moves beyond sample averages to reveal how these constructs naturally cluster within distinct profiles of learners. The identification of four distinct latent profiles—ranging from optimal functioning to severe distress—unveils a substantial heterogeneity within the Chinese EFL writing population (RQ2), offering a more granular ecological map of learner psychology.

Over half of the sample exhibited adaptive profiles, yet with a crucial distinction in intensity. The High Efficacy and Enjoyment Profile epitomizes the ideal psychological state of “flourishing” described in Positive Psychology (Dewaele and Li, 2020). Characterized by high self-efficacy and enjoyment alongside minimal negative affect, their higher engagement across both dimensions confirms that when learners feel fully competent and intrinsically motivated, they enter a virtuous cycle of deep processing and sustained effort. In contrast, the Moderate Efficacy and Enjoyment Profile represents the “typical” learner. While functionally adaptive, their motivation is less robust. The sheer size of this group suggests that the norm in EFL writing in the current study is a state of moderate competence that lacks the high-intensity positive affect required for optimal performance. This distinction underscores that low levels of distress do not automatically equate to the presence of thriving.

The remaining 44% of students fell into maladaptive categories, highlighting pervasive affective and efficacy-related challenges. The Low Efficacy and Moderate Negative Affect Profile constitutes a large, arguably “at-risk” subgroup. These students are defined by a confidence deficit (low efficacy) rather than high levels of negative affect. Their profile suggests a state of “disengaged coping,” where low belief in one’s capabilities suppresses enjoyment and invites moderate levels of anxiety and boredom. Most critically, the Lowest Efficacy and High Distress Profile reveals a severe “crisis” group. Theoretically, this profile offers a significant insight: while CVT classifies anxiety as “activating” (high arousal) and boredom as “deactivating” (low arousal) (Pekrun, 2006), this classification does not preclude their co-occurrence within the same individual. This profile likely reflects a state of complete “loss of control” (lowest efficacy), where the learner is simultaneously overwhelmed by the fear of failure (anxiety) and emotionally detached from the meaningless task (boredom). This pattern of emotional co-occurrence represents a level of complexity that variable-centered analyses would be unlikely to capture.

The predictive validity of these profiles was supported by the engagement data (RQ3). The results demonstrated a clear hierarchical pattern across the cognitive dimension, corroborating the central tenet of CVT that emotional experiences are correlates of engagement quality. However, a nuanced finding emerged regarding behavioral engagement. While the “High Distress” group generally scored the lowest, their behavioral effort was statistically indistinguishable from the “Low Efficacy” group. This suggests behavioral engagement may be comparatively less sensitive to differences in emotional intensity than cognitive engagement, once self-efficacy falls to a low level. This exploratory observation implies that behavioral withdrawal may be more closely tied to the loss of efficacy than to the specific emotional profile accompanying it, reinforcing the variable-centered finding of self-efficacy as the primary driver—though this interpretation warrants confirmation.

### 5.3 Synthesis: the dialogue between universality and specificity

The synthesis of variable-centered and person-centered findings yields an informative dialogue between the structural generality of SLA models and the contextual specificity of L2 writers. Rather than presenting disparate accounts, the two approaches appear to complement each other by illustrating how general psychological associations may manifest differently within heterogeneous learner sub-populations.

On one hand, the variable-centered pathways are consistent with the general applicability of the Control-Value Theory. The structural model suggests that the core associative sequence of CVT—where control appraisals (WSE) are linked to achievement emotions, which in turn are associated with engagement—appears to hold within the specific domain of Chinese tertiary EFL writing. The roles of boredom as a correlate of behavioral engagement and enjoyment as a correlate of cognitive engagement are consistent with the cross-contextual relevance of CVT, lending support to the broader proposition that the associative architecture linking affect and engagement may operate similarly across diverse learning contexts.

On the other hand, the person-centered configuration reveals a layer of specificity that qualifies these sample-average patterns. While the variable-centered model indicates that anxiety can paradoxically act as an activating emotion associated with cognitive effort when other factors are controlled, the latent profile analysis illustrates how anxiety functions within real-world learner profiles. For 44% of the sample, anxiety co-occurred with substantial boredom and a pronounced confidence deficit, a configuration associated with lower engagement overall. The most vulnerable group appeared to reflect a state in which low-arousal and high-arousal negative emotions co-occurred alongside severely diminished self-efficacy, and this profile exhibited the poorest engagement among all groups. This pattern of emotional co-occurrence illustrates a level of contextual complexity that variable-centered analyses, which operate on sample-level averages, would be unlikely to capture.

Furthermore, the synthesis points to a converging pattern regarding the role of writing self-efficacy. In the structural model, WSE showed a significant direct association with engagement independent of emotional mediation. A related pattern emerged in the profile analysis: once a learner’s self-efficacy falls to a low level, their behavioral engagement appears similarly low, regardless of whether they experience moderate negative affect or more severe distress. This complementarity between analytical perspectives suggests that efficacy beliefs function as an essential prerequisite for writing engagement, working in tandem with emotional experiences to shape students’ overall investment in writing.

### 5.4 Implications

Theoretically, this study provides empirical support for the Control-Value Theory as a useful framework for understanding L2 writing psychology. The significant direct paths from self-efficacy to both behavioral and cognitive engagement, alongside its indirect associations through emotional pathways, suggest that control appraisals may be linked to engagement both directly and through achievement emotions, supporting a partial rather than full mediation account. This pattern implies that interventions targeting emotions alone may be insufficient without also addressing learners’ fundamental belief in their writing capability. Furthermore, the differentiated mediating roles of the three emotions suggest that the associations between emotions and engagement may depend on which dimension of engagement is considered, rather than operating uniformly across all forms of investment.

The findings of this study suggest the potential value of moving beyond one-size-fits-all instruction toward differentiated instructional consideration within the Chinese EFL writing context. Given that self-efficacy showed a direct association with engagement, a primary pedagogical consideration may be efficacy building for the broader learner population. Instructors may consider prioritizing mastery experiences by breaking complex writing tasks into manageable micro-goals. Providing informational feedback that attributes success to effort and strategy use—rather than vague praise—may help support the development of self-efficacy among students. Given that boredom emerged as the consistent correlate of behavioral engagement, instructors might also consider incorporating more varied and personally relevant writing tasks to mitigate behavioral withdrawal. As for more targeted support, students in the Lowest Efficacy and High Distress profile may benefit from psychologically safe learning environments, as high-stakes pressure may exacerbate their co-occurring anxiety and boredom. Instructors might consider implementing low-stakes writing practices and introducing explicit emotion regulation strategies. Given the nuanced association observed between anxiety and cognitive engagement, such strategies should be carefully calibrated rather than aimed solely at minimizing anxiety.

## 6 Conclusion

Drawing on the Control-Value Theory, the present study examined how writing self-efficacy is associated with behavioral and cognitive engagement, both directly and through the differentiated mediating roles of enjoyment, anxiety, and boredom, while also identifying distinct learner profiles characterized by varying configurations of efficacy and emotion. By integrating variable-centered and person-centered perspectives, this dual-lens approach offers a more complete account of the psychological landscape underlying L2 writing engagement than either approach could provide alone.

The findings make two principal contributions. First, they extend CVT to the domain of L2 writing by demonstrating that self-efficacy is associated with engagement through pathways that differ across emotion and engagement dimensions, challenging the assumption that emotional mediation operates uniformly across all forms of engagement. Second, by revealing four distinct learner profiles, the study illustrates a level of individual heterogeneity that average-based analyses would be unlikely to capture, underscoring the value of considering both general associative patterns and learner-specific variation in future L2 writing research.

Despite these contributions, several limitations warrant consideration. First, the cross-sectional design precludes causal inferences. While the CVT framework implies a causal sequence, longitudinal or experimental designs are needed to more directly examine these temporal dynamics. Second, the reliance on self-report measures, though standard in the field, may be subject to social desirability bias or limits in learners’ emotional granularity. Future research could benefit from integrating physiological measures or qualitative interviews to capture the “lived experience” of learners. Third, the operationalization of writing engagement in the present study was limited to behavioral and cognitive dimensions, following the exclusion of affective engagement due to its conceptual proximity to the emotion constructs. While this decision preserved discriminant validity, it inevitably narrowed the scope of the engagement construct. Future research could develop or adopt engagement measures with cleaner boundaries from achievement emotion scales to enable a more comprehensive assessment. Finally, as the study was situated within the specific context of Chinese tertiary EFL writing, caution should be exercised when generalizing these findings to other cultural or educational settings.

## Data Availability

The raw data supporting the conclusions of this article will be made available by the authors, without undue reservation.
